# Rehabilitation for patients with sepsis: A systematic review and meta-analysis

**DOI:** 10.1371/journal.pone.0201292

**Published:** 2018-07-26

**Authors:** Shunsuke Taito, Mahoko Taito, Masahiro Banno, Hiraku Tsujimoto, Yuki Kataoka, Yasushi Tsujimoto

**Affiliations:** 1 Division of Rehabilitation, Department of Clinical Practice and Support, Hiroshima University Hospital, Hiroshima, Japan; 2 Department of Nursing, Hiroshima University Hospital, Hiroshima, Japan; 3 Department of Psychiatry, Seichiryo Hospital, Nagoya, Aichi, Japan; 4 Department of Psychiatry, Nagoya University Graduate School of Medicine, Nagoya, Aichi, Japan; 5 Hospital Care Research Unit, Hyogo Prefectural Amagasaki General Medical Center, Hyogo, Japan; 6 Department of Respiratory Medicine, Hyogo Prefectural Amagasaki General Medical Center, Hyogo, Japan; 7 Department of Healthcare Epidemiology, School of Public Health, Graduate School of Medicine, Kyoto University, Kyoto, Japan; 8 Department of Nephrology and Dialysis, Kyoritsu Hospital, Hyogo, Japan; Hospital Universitari Bellvitge, SPAIN

## Abstract

The objective of this systematic review was to determine whether rehabilitation impacts clinically relevant outcomes among adult patients with sepsis. Randomized controlled trials from the Cochrane Central Register of Controlled Trials (CENTRAL), MEDLINE, EMBASE, Cumulative Index to Nursing and Allied Health Literature (CINAHL), PEDro, and the World Health Organization International Clinical Trials Platform Search Portal, as well as conference proceedings and reference lists of relevant articles were collected. Two reviewers independently identified randomized controlled trials on the rehabilitation of patients with sepsis, and the two reviewers independently abstracted trial level data including population characteristics, interventions, comparisons, and clinical outcomes. Our primary outcomes were quality of life (QOL), activity of daily living (ADL), and mortality. Our secondary outcomes were length of stay, return to work, muscle strength, delirium, and all adverse events. The quality of evidence was determined using the Grading of Recommendations Assessment, Development, and Evaluation (GRADE) approach. We included two trials enrolling 75 patients. The mean difference (95% confidence interval [CI]) of physical function and physical role in QOL measured by SF-36 were 21.10 (95% CI: 6.57–35.63) and 44.40 (95% CI: 22.55–66.05), respectively. Rehabilitation did not significantly decrease intensive care unit (ICU) mortality (risk ratio, 2.02 [95% CI: 0.46–8.91], *I*^2^ = 0%; n = 75). ICU length of stay and hospital length of stay and muscle strength were not statistically significantly different and no adverse events were reported in both studies. The certainty of the evidence for these outcomes was “very low.” Data on ADL, return to work, and delirium were not available in any of the trials. Rehabilitation of patients with sepsis might not decrease ICU mortality, but might improve QOL. Further, well-designed trials measuring important outcomes will be needed to determine the benefit and harm of rehabilitation among patients with sepsis.

## Introduction

Sepsis is a major healthcare problem, affecting millions of people around the world each year [1]. Sepsis was identified in 6.0% of all adult patients admitted to hospitals in the United States, among whom 15.0% died on admission and 6.2% were discharged to hospice care [2]. The readmission rate in patients with sepsis following discharge was reported as 35% within 6 months and 60% within a year [3, 4]. It has also been reported that after discharge, one-third of patients with a diagnosis of sepsis had not returned to independent living after 6 months [5]. Furthermore, the long-term health-related quality of life (QOL) outcomes in such patients were poorer than in other populations [6, 7]. Guidelines recommend that critically-ill patients undergo rehabilitation in an intensive care unit (ICU) [8, 9]. Recent randomized controlled studies have reported that rehabilitation reduced the incidence of delirium, and improved physical function and activities of daily living (ADL) in ICU patients [10]. Rehabilitation also provides additional advantages, including improvement in psychological and cognitive functions, promotion of social participation, and increase in the opportunity to return to work [11–13]. Physical rehabilitation is an important strategy for enhancing recovery from critical illness and for improving the symptoms of post-intensive care syndrome (PICS) [13].

In a recent systematic review and meta-analysis [14] of critically-ill patients, rehabilitation had no impact on QOL and mortality. A pilot randomized controlled trial (RCT) of patients with sepsis in ICU reported that rehabilitation improved QOL [15], and a larger effect of rehabilitation may be expected for such patients with sepsis. This review [14] only included trials in ICU settings. However, patients with sepsis are also treated in non-ICU wards, and the classification of wards differs among countries [16]. One of the goals of the Global Sepsis Alliance [17] is “ensuring improved access to adequate rehabilitation services” [18]. However, no systematic reviews and meta-analyses have yet reported the effect of rehabilitation in patients with sepsis. Therefore, the objective of our study was to determine whether rehabilitation impacts clinically relevant outcomes, compared with usual care, among adult patients with sepsis.

## Materials and methods

Using a pre-specified published protocol (PROSPERO: CRD42017076384) [19], we conducted a systematic review based on the Cochrane Handbook [20] and the Preferred Reporting Items for Systematic Reviews and Meta-Analysis guidelines [21]. We assessed this systematic review using the PRISMA 2009 checklist [22]. An additional file shows this in more detail (S1 File. PRISMA 2009 Checklist).

### Research question and eligibility criteria

We posed the following research question: “In adult patients with sepsis, does rehabilitation compared with usual care result in improved clinically relevant outcomes?” We included all published and unpublished prospective RCTs in human subjects who were adults (aged ≥18 years) with sepsis (defined by the international guideline [23–26] or authors’ definitions, including those that were only abstracts or letters. Crossover trials and cluster-, quasi-, or non-randomized trials were excluded. Studies were included regardless of the time of follow-up. We included patients of any sex, race, and setting, and excluded those with head or spinal cord injury, and unstable fractures contributing to probable immobility. The intervention in the review was protocolized rehabilitation in hospitals, including neuromuscular stimulation, passive range of motion exercise, or active exercises, designed to either commence earlier and/or be more intensive than the care received by the control group. Any combination of one or more of the following were considered: neuromuscular stimulation, inspiratory or respiratory muscle training, passive range of motion exercise, cycle ergometry, active-assisted exercises, mobility activities in bed, ADLs, transfer, marching on the spot, and walking exercise. Our primary outcomes were QOL, ADL assessed using standard measures such as function independence measures and the Barthel index, and mortality. Secondary outcomes were length of stay (ICU and hospital), return to work, muscle strength, delirium, and all adverse events (defined by the trialists).

### Search strategy and selection of studies

We searched the Cochrane Central Register of Controlled Trials (CENTRAL), MEDLINE via Ovid, EMBASE via Elsevier, PEDro, and the World Health Organization International Clinical Trials Registry Platform (WHO ICTRP) on September 18, 2017. We re-ran the search using the Cumulative Index to Nursing and Allied Health Literature (CINAHL) via EBSCO on May 26, 2018. An additional file shows this process in more detail (S2 File. Search Strategy). We also hand-searched reference lists for the latest guidelines on sepsis [26], as well as reference lists of extracted studies and articles citing extracted studies, using the Web of Science. We contacted authors of extracted studies if these studies lacked necessary information. Two reviewers (ST and MT) independently screened titles and abstracts of search results to determine whether each citation met the inclusion criteria. They assessed the eligibility based on a full-text review. Disagreement was resolved by discussion, and if necessary, YT was brought in for arbitration.

### Data abstraction and quality assessment

Two reviewers (ST and MT) also independently abstracted trial level data using pre-specified forms. Disagreements in data extraction were resolved through discussions. We contacted authors of studies without sufficient information where necessary. Two reviewers (ST and MT) independently assessed the risk of bias in the included studies using the Cochrane Risk of Bias Assessment Tool [20]. Differences in opinion following the assessment of risk of bias were resolved through discussions, and where this failed, through arbitration by YT.

### Data analysis

All analyses were conducted using the Review Manager software (RevMan 5.3; Cochrane Community). We pooled the risk ratio (RR) with 95% confidence interval (CI) for the dichotomous variables: mortality and return to work. For continuous outcomes—QOL, ADL, length of stay (ICU and hospital), muscle strength, delirium days (ICU and hospital), the standardized mean difference (SMD), or mean differences (MD) with 95% CI were calculated, as recommended by the Cochrane Handbook [18]. Adverse events were narratively summarized because the definition of these outcomes varied from study to study. We used the random-effects models for all analyses. To explore potential heterogeneity and determine whether the level of risk of bias affected the effect estimate, we planned to conduct subgroup and sensitivity analyses. However, the included studies were insufficient to perform these analyses. We investigated the reporting bias by checking the trial registers (WHO ICTRP) and detected completed but unpublished trials. P<0.05 was considered statistically significant. The summary of findings is presented in Table 1, which includes an overall grading of the evidence using the Grading of Recommendations, Assessment, Development, and Evaluation (GRADE) approach [27, 28].

**Table 1:** Summary of findings.

Outcome	Illustrative comparative risks* (95% CI)		Relative effect (95% CI)	No. of participants (studies)	Certainty of the evidence (GRADE)	Comments
	Risk usual care	Risk rehabilitation				
**Quality of life** SF-36 (at 6 months)	Mean difference [95% CI] of physical function and physical role were 21.10 [6.57–35.63] and 44.40 [22.55–66.05] respectively. These mean differences were significantly higher for those who received intervention.		-	30 (1 RCT)	⊕⊝⊝⊝ **Very low** ^a^ ^b^ ^c^	
**ICU mortality**	Study population		RR 2.02 (0.46 to 8.91)	75 (2 RCT)	⊕⊝⊝⊝ **Very low** ^b^ ^c^	
	65 per 1,000	130 per 1,000 (30 to 575)				
**ICU length of stay**	Median (interquartile range) of ICU length of stay was not statistically significantly different in both studies. Intervention vs. comparison: 12 (4–45) vs. 8.5 (3–36) days		-	50 (1 RCT)	⊕⊝⊝⊝ **Very low** ^a^ ^b^ ^c^	
**Hospital length of stay**	Hospital length of stay was not statistically significantly different in both studies. Intervention vs. comparison: 41 (9–158) vs. 45 (14–308) days and 30 (18–45) vs. 36 (26–78) days		-	75 (2 RCT)	⊕⊝⊝⊝ **Very low** ^a^ ^b^ ^c^	
**Muscle strength** MRC sum-score (at ICU discharge)	Mean difference [95% CI] of MRC sum-score was 4.6 [-2.69–11.89]. The mean difference was higher for those who received intervention.		-	42 (1 RCT)	⊕⊝⊝⊝ **Very low** ^a^ ^b^ ^c^	
**Adverse events**	Two studies reported no adverse events.		-	75 (2 RCT)	⊕⊝⊝⊝ **Very low** ^a^ ^b^ ^c^	

*The corresponding risk (and its 95% confidence interval) is based on the assumed risk in the comparison group and the relative effect of the intervention (and its 95% CI).

CI: confidence interval; RR: risk ratio

GRADE Working Group grades of evidence

**High certainty**: We are very confident that the true effect lies close to that of the estimate of the effect.

**Moderate certainty**: We are moderately confident in the effect estimate: The true effect is likely to be close to the estimate of the effect, but there is a possibility that it is substantially different.

**Low certainty**: Our confidence in the effect estimate is limited: The true effect may be substantially different from the estimate of the effect.

**Very low certainty**: We have very little confidence in the effect estimate: The true effect is likely to be substantially different from the estimate of the effect.

^*a*^ Participants and personnel were not blinded.

^*b*^ Number of participants was small.

^*c*^ There were four ongoing studies.

## Results

### Study selection, characteristics and quality

**Fig 1:**
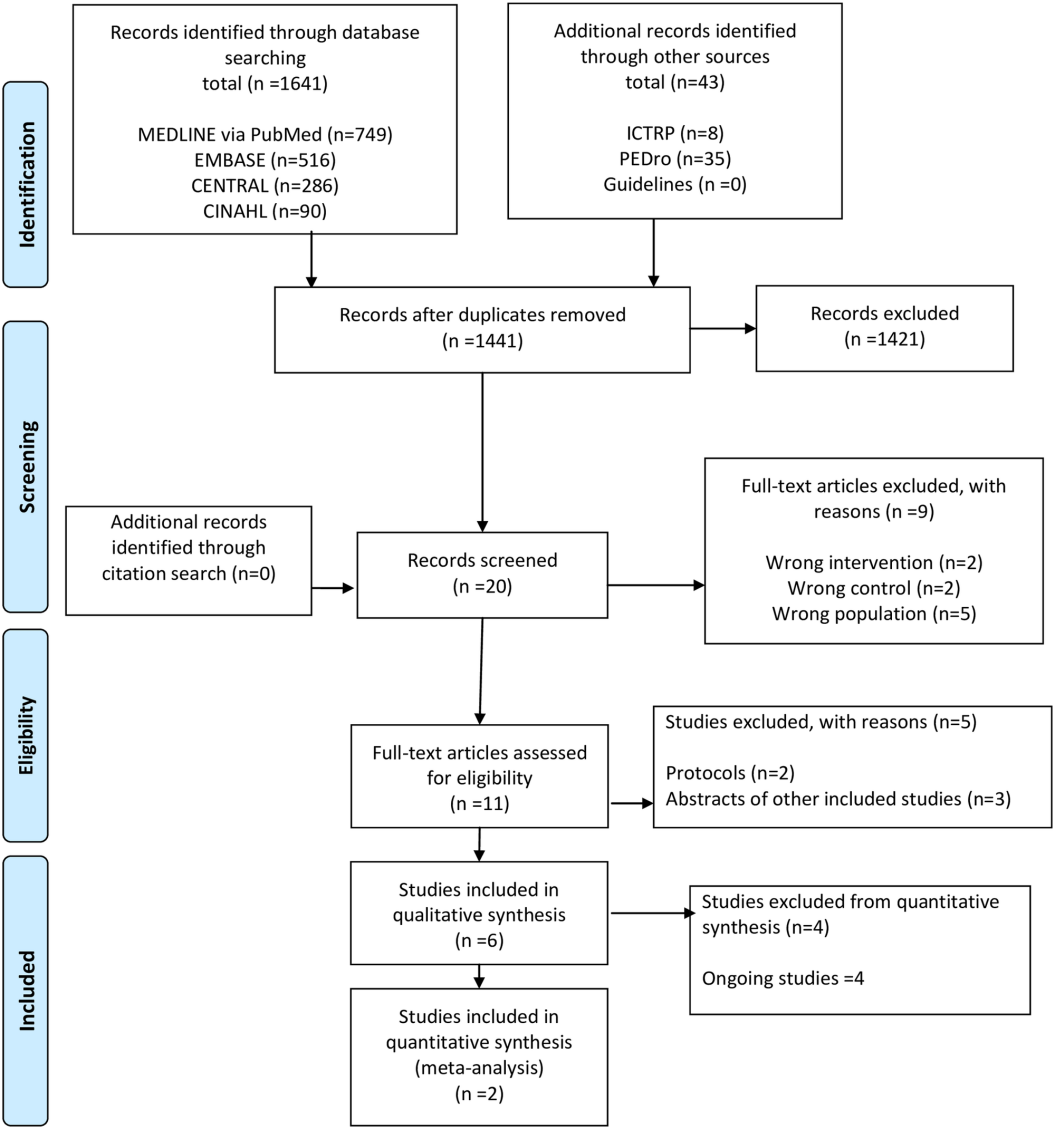
Preferred reporting items for systematic reviews and meta-analyses (PRISMA) flow diagram.

Of 1,684 citations retrieved, we included two unique RCTs (Fig 1 and Table 2) [15, 29]. In total, 75 patients were represented across the two studies (44 interventions and 31 controls). Both trials evaluated the effect of rehabilitation in an ICU setting. The median ages of the patients were 62.5 (inter-quartile range [IQR]: 30–83) and 77.5 (72–81) years, respectively. The mean Acute Physiology and Chronic Health Evaluation (APACHE) II score was 28.0 (standard deviation [SD]: 7.6) and median: 23.5 (IQR: 19–28). The interventions in the included studies were provided over 5 times per week and ranged from 30 to 60 min of therapy per day in ICUs. These interventions mainly involved passive or active assisted exercise and neuromuscular stimulation.

**Table 2:** Characteristics of included studies.

Author, Year, Country	Setting	No of participants	Study type	Intervention (contents, frequency)	Control (standard care)	Outcomes	Note
Kayambu G et al., 2015, Australia	ICU	50 (intervention: 26, control: 24)	Pilot RCT	Intervention for 30 min, one to two times daily until discharge from the ICU within 48 h of the diagnosis of sepsis. Physical rehabilitation strategies included electrical muscle stimulation, passive range of motion, active range of motion, sitting out of bed, transfers, ambulation and other mobilization techniques.	Usual care (standard ICU care which included physical therapy strategies provided by the ICU physiotherapist).	Physical function (acute care index of function) and self-reported health-related quality of life) at ICU discharge and 6 months posthospital discharge, inflammatory biomarkers (interleukin-6, interleukin-10 and tumor necrosis factor-α), blood lactate, fat free muscle mass, exercise capacity, muscle strength and anxiety, ICU mortality, 30-day mortality, ICU length of stay, hospital length of stay, and adverse events.	The protocol is published (BMC Anesthesiol. 2011 Oct 31;11:21) Trial ID: ACTRN 12610000808044
Shen SY et al., 2017, Taiwan	ICU	25 (intervention: 18, control: 7)	Pilot RCT	In addition to usual care, electrical muscle stimulation on both quadriceps (vastus medialis) and biceps, 32 minutes per day, 5 days per week (Monday to Friday) until discharge from the ICU after patients required mechanical ventilation longer than 72 hours.	Usual care (active or passive exercise of extremities which was decided and performed by the physical rehabilitation therapist after consultation).	ICU mortality*, hospital mortality, mechanical ventilation days, successful spontaneous breathing, and adverse events.	Trial ID: NCT01895647A

*Unpublished data

We identified four studies as ongoing and confirmed the trial status through direct contact with the lead authors. The characteristics of these ongoing studies, including details of participants, interventions, control group, and outcomes are summarized in an additional file (S1 Table. Characteristics of ongoing studies). Briefly, participants included from ongoing studies were patients diagnosed with sepsis, severe sepsis, or septic shock. The interventions included additional physical therapy including passive/active cycling exercise, functional electrical stimulation, and early mobilization. QOL, ADL, mortality, length of stay in hospital or ICU, employment status, and muscle strength were included as outcomes in these trials. No further articles were found from hand searches.

The risk of bias assessment is outlined in Table 3. In both studies, participants and personnel were not blinded to the intervention. Also, the studies had incomplete outcomes and selective reporting. Shen’s study reported unknown risk of bias from published data, and as such, we contacted the authors. According to the authors, nurses not part of the study drew lots and the investigator was not aware of the randomization sequence, but there was no blinding of the outcome assessment. One study was funded by the Intensive Care Foundation.

**Table 3:** Assessment of risk of bias in included trials.

Trial	Random sequence generation	Allocation concealment	Blinding of participants and personnel	Blinding of outcome assessment	Incomplete outcome data	Selective reporting	Other biases
Kayambu et al. [15]	Low	Low	High	Low	High	High	Low
Shen et al. [29]	Low	Low	High	High	High	High	Unknown*

*: Very little detail given regarding the therapy received in the control group

The main results of our review are presented in Table 1.

### Primary outcomes

**Fig 2:**
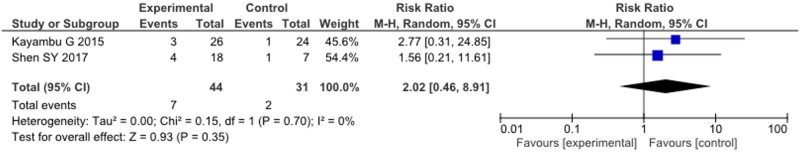
Effect of rehabilitation on ICU mortality.

Data on QOL were available in 1 of the 2 trials [15], while data on ADL were not available in any of the trials. ICU mortality was reported in both trials [15, 29]. Mean difference (95% CI) of physical function and physical role in QOL were 21.10 (6.57–35.63) and 44.40 (22.55–66.05), respectively. Rehabilitation did not significantly decrease ICU mortality (RR, 2.02; 95% CI, 0.46–8.91, *I*^2^ = 0%; n = 75) (Fig 2). The certainty of the evidence for QOL and ICU mortality were “very low” (Table 1).

### Secondary outcomes

There was no significant differences in ICU length of stay and hospital length of stay between the intervention and control groups in both studies [15, 29]. The data on muscle strength were available in 1 of 2 trials [15], and the mean difference (95% CI) of MRC sum-score was 4.60 (-2.69–11.89) and higher among those who received intervention.

Data on return to work and delirium were not available in any of the trials. Both studies reported no adverse event in both the intervention and standard care groups.

## Discussion

We observed no reduction in ICU mortality among patients with sepsis who received rehabilitation. Rehabilitation could improve QOL but did not improve hospital length of stay or muscle strength, although data for these outcomes were limited. Studies on the rehabilitation of patients with sepsis are very few, and the certainty of the evidence was very low, from the findings of this systematic review.

Rehabilitation of patients with sepsis might improve QOL but might not decrease ICU mortality compared with standard care based on two studies which had wide confidence intervals and small sample sizes. This was different from a recent systematic review of critically-ill patients [14] which reported that the mean difference (95% CI) of physical function and physical role in QOL measured by SF-36 were 6.44 (-4.57–417.45) and 17.33 (-13.10–147.76), respectively and the risk difference (95% CI) of ICU mortality from eight studies was 0.02 [-0.01–0.05]. The review [14] determined the effect of rehabilitation in ICU among patients regardless of disease and our review was like a subgroup analysis of the review. Sepsis is a high risk factor for PICS such as ICU-acquired weakness and delirium [30, 31], and is associated with high mortality [2] and readmission rates [3, 4]. Physical rehabilitation is an important strategy for enhancing the recovery of critically-ill patients and for addressing the symptoms of PICS [13]. Thus, we thought that sepsis patients respond to rehabilitation differently and might truly benefit from physical rehabilitation. Rehabilitation might improve QOL for only sepsis patients in the ICU although the pilot studies had high risks of bias. On the other hand, some studies for other illnesses, including the exacerbation of chronic respiratory disease [32] and stroke [33] reported that early intensive rehabilitation may increase mortality and unfavorable outcomes. There are several ongoing studies, and further studies with low risk of bias are needed to further investigate this.

The main purpose of rehabilitation in the ICU is to improve QOL by maintaining, improving, and reacquiring ADL [34], including returning to work for survivors after hospital discharge. Sepsis is a risk factor for PICS [13] such as ICU-acquired weakness [35] and delirium [36] which are associated with QOL [37, 38]. Thus, we selected these outcomes to determine the effect of rehabilitation in patients with sepsis; however, ADL and delirium were not reported. Recently, the need for consensus on core outcomes set for patients with sepsis was agreed upon [39], and medical staff, patients, and caregiver representatives set core outcomes for acute respiratory failure survivors [40, 41]. Ongoing studies investigating ADL and employment status [42] will reveal the core outcomes needed for patients with sepsis, with respect to rehabilitation after hospital discharge, including ADL, delirium, and returning to work [43].

Generalizability of rehabilitation for patients with sepsis in ICU may be poor. Only approximately 5% of patients screened were included [15, 29] and there were also large differences between the number of participants recruited and the planned recruitment. The large difference between the number of participants recruited and those originally planned to be recruited was not only a potential feasibility issue in conducting trials in patients with sepsis but also the timing of the commencement of the intervention in which patients were managed by mechanical ventilation. Both reviewed studies included neuromuscular stimulation and needed stimulators for intervention. Thus, not all facilities could provide the intervention. Secondly, intensive rehabilitation could increase work burden for physicians, nurses, and physical therapists. The interventions in the included studies started in the ICU, and highly professional interventions based on adequate assessment were needed for critically-ill patients in the ICU. Except for facilities with adequate well-trained human resources, there are concerns about the feasibility of rehabilitation in the ICU. There is the need for clinical trials about more feasible interventions.

This review did not include trials in non-ICU settings because the reviewed studies were only conducted in ICU settings. A few studies reported on the rehabilitation of critically-ill patients after ICU discharge [44], and an ongoing study is examining the effect of intervention after hospital discharge [45]. However, there might be differences in the effect of rehabilitation on patients with low disease severity, and further studies are needed to examine the effect of rehabilitation in non-ICU settings.

This systematic review has potential limitations. We only included two RCTs in this review, and we might not have searched exhaustively. However, we tried to enhance the comprehensiveness in this review by identifying more studies by searching other sources and performing citation search. The trials were at high risk of bias due to the lack of blinding in the intervention arms, which may have contributed to performance bias. Mortality might not be affected by performance bias, but other outcomes such as the QOL and muscle strength could be affected by the bias.

## Conclusions

Rehabilitation of patients with sepsis might not decrease ICU mortality nor improve the length of stay in hospital and muscle strength, but could improve QOL compared with standard care based on two studies which had wide confidence intervals and small sample sizes. However, data for these outcomes were limited and future well-designed prospective randomized controlled trials with a low risk of bias to provide definitive conclusions on this topic are needed.

## Supporting information

S1 File PRISMA 2009 checklist. (DOC)

S2 File Search strategy. (DOCX)

S1 Table Characteristics of ongoing studies. (DOCX)
