# Correction: Rehabilitation for patients with sepsis: A systematic review and meta-analysis

**DOI:** 10.1371/journal.pone.0221224

**Published:** 2019-08-21

**Authors:** Shunsuke Taito, Mahoko Taito, Masahiro Banno, Hiraku Tsujimoto, Yuki Kataoka, Yasushi Tsujimoto

There is a numerical error that appears in the Abstract, Table 1, and the Results. The mean difference (95% confidence interval (CI)) of physical function and physical role in quality of life (QOL) incorrectly appears as 21.10 (95% CI: 6.57–35.63) and 44.40 (95% CI: 22.55–66.05), respectively. The correct mean difference (95% confidence interval (CI)) of physical function and physical role in quality of life (QOL) were 21.80 (3.18–40.42) and 44.30 (14.15–74.45). Please see the correct Table 1 below.

**Table 1:** Summary of Findings.

Rehabilitation compared with usual care in adult patients with sepsis						
Patients or study population: adult patients with sepsis Setting: any Intervention: protocolized rehabilitation designed to either commence earlier, and/or more intensive than the care received by the control group Comparison: usual care						
Outcome	Illustrative comparative risks* (95% CI)		Relative effect (95% CI)	No. of participants (studies)	Certainty of the evidence (GRADE)	Comments
	Risk usual care	Risk rehabilitation				
**Quality of life** SF-36 (at 6 months)	Mean difference [95% CI] of physical function and physical role were 21.80 [3.18–40.42] and 44.30 [14.15–74.45] respectively. These mean differences were significantly higher for those who received intervention.		-	30 (1 RCT)	⊕⊝⊝⊝ **Very low** ^a^ ^b^ ^c^	
**ICU mortality**	Study population		RR 2.02 (0.46 to 8.91)	75 (2 RCT)	⊕⊝⊝⊝ **Very low** ^b^ ^c^	
	65 per 1,000	130 per 1,000 (30 to 575)				
ICU length of stay	Median (interquartile range) of ICU length of stay was not statistically significantly different in both studies. Intervention vs. comparison: 12 (4–45) vs. 8.5 (3–36) days		-	50 (1 RCT)	⊕⊝⊝⊝ **Very low** ^a^ ^b^ ^c^	
**Hospital length of stay**	Hospital length of stay was not statistically significantly different in both studies. Intervention vs. comparison: 41 (9–158) vs. 45 (14–308) days and 30 (18–45) vs. 36 (26–78) days		-	75 (2 RCT)	⊕⊝⊝⊝ **Very low** ^a^ ^b^ ^c^	
**Muscle strength** MRC sum-score (at ICU discharge)	Mean difference [95% CI] of MRC sum-score was 4.6 [-2.69–11.89]. The mean difference was higher for those who received intervention.		-	42 (1 RCT)	⊕⊝⊝⊝ **Very low** ^a^ ^b^ ^c^	
Adverse events	Two studies reported no adverse events.		-	75 (2 RCT)	⊕⊝⊝⊝ **Very low** ^a^ ^b^ ^c^	
*The corresponding risk (and its 95% confidence interval) is based on the assumed risk in the comparison group and the relative effect of the intervention (and its 95% CI). CI: confidence interval; RR: risk ratio						
**GRADE Working Group grades of evidence** **High certainty**: We are very confident that the true effect lies close to that of the estimate of the effect. **Moderate certainty**: We are moderately confident in the effect estimate: The true effect is likely to be close to the estimate of the effect, but there is a possibility that it is substantially different. **Low certainty**: Our confidence in the effect estimate is limited: The true effect may be substantially different from the estimate of the effect. **Very low certainty**: We have very little confidence in the effect estimate: The true effect is likely to be substantially different from the estimate of the effect.						

^*a*^ Participants and personnel were not blinded.

^*b*^ Number of participants was small.

^*c*^ There were four ongoing studies.

Additionally, there are citation errors in the Methods section. The fourth sentence of the third paragraph should have cited reference 25 instead of 26. The third sentence of the fifth paragraph should have cited reference 20 instead of 18.
