# Supplementary material for: Rehabilitation for patients with sepsis: A systematic review and meta-analysis
Source: PLoS One. 2018 Jul 26;13(7):e0201292. doi: 10.1371/journal.pone.0201292 (PMC6062068; doi:10.1371/journal.pone.0201292)
Supplement: S2 File — (DOCX) [file pone.0201292.s002.docx]

**The cochrane central register of controlled trials (CENTRAL)**

#1 MeSH descriptor:[Systemic Inflammatory Response Syndrome]explode all trees

#2 "systemic inflammatory response syndrome":ti,ab,kw

#3 SIRS:ti,ab,kw

#4 sepsis:ti,ab,kw

#5 septic:ti,ab,kw

#6 #1 OR #2 OR #3 OR #4 OR #5

#7 MeSH descriptor:[Rehabilitation]explode all trees

#8 MeSH descriptor:[Exercise]explode all trees

#9 #7 OR #8

#10 rehabilitation:ti,ab,kw

#11 exercise:ti,ab,kw

#12 training:ti,ab,kw

#13 mobilization:ti,ab,kw

#14 mobilisation:ti,ab,kw

#15 "physical therapy":ti,ab,kw

#16 physiotherapy:ti,ab,kw

#17 "occupational therapy":ti,ab,kw

#18 "electrical muscle stimulation":ti,ab,kw

#19 cycle ergometer:ti,ab,kw

#20 bridging:ti,ab,kw

#21 rolling:ti,ab,kw

#22 "lying to sitting":ti,ab,kw

#23 marching:ti,ab,kw

#24 ambulation:ti,ab,kw

#25 "activities of daily living":ti,ab,kw

#26 ADL:ti,ab,kw

#27 walking:ti,ab,kw

#28 #9 OR #10 OR #11 OR #12 OR #13 OR #14 OR #15 OR #16 OR #17 OR # 18 OR #19 OR # 20 OR #21 OR # 22 OR #23 OR #24 OR #25 OR #26 OR #27

#29 #6 AND #28

**MEDLINE via Ovid**

#1 exp Systemic Inflammatory Response Syndrome/

#2 Systemic inflammatory response syndrome.mp.

#4 SIRS.mp.

#4 sepsis.mp.

#5 septic.mp.

#6 or/1-5

#7 exp Rehabilitation/

#8 exp Exercise/

#9 7 or 8

#10 rehabilitation.mp.

#11 exercise.mp.

#12 training.mp.

#13 mobilization.mp.

#14 physical therapy.mp.

#15 physiotherapy.mp.

#16 occupational therapy.mp.

#17 electrical muscle stimulation.mp.

#18 cycle ergometer.mp.

#19 bridging.mp.

#20 rolling.mp.

#21 lying to sitting.mp.

#22 marching.mp.

#23 ambulation.mp.

#24 activities of daily living.mp.

#25 ADL.mp.

#26 walking.mp.

#27 or/10-26

#28 9 or 27

#29 randomized controlled trial.pt.

#30 controlled clinical trial.pt.

#31 randomized.ab.

#32 placebo.ab.

#33 drug therapy.fs.

#34 randomly.ab.

#35 trial.ab.

#26 groups.ab.

#37 or/29-36

#38 exp animals/ not humans.sh.

#39 37 not 38

#40 6 and 28 and 39

**EMBASE**

#1 "systemic inflammatory response syndrome"/exp

#2 "systemic inflammatory response syndrome":ab,ti

#3 SIRS:ab,ti

#4 sepsis:ab,ti

#5 septic:ab,ti

#6 #1 OR #2 OR #3 OR #4 OR #5

#7 Rehabilitation/exp

#8 Exercise/exp

#9 #7 OR #8

#10 rehabilitation:ab,ti

#11 exercise:ab,ti

#12 training:ab,ti

#13 mobilization:ab,ti

#14 "physical therapy":ab,ti

#15 physiotherapy:ab,ti

#16 "occupational therapy":ab,ti

#17 "electrical muscle stimulation":ab,ti

#18 "cycle ergometer":ab,ti

#19 bridging:ab,ti

#20 rolling:ab,ti

#21 "lying to sitting":ab,ti

#22 marching:ab,ti

#23 ambulation:ab,ti

#24 "activities of daily living":ab,ti

#25 ADL:ab,ti

#26 walking:ab,ti

#27 #10 OR #11 OR #12 OR #13 OR #14 OR #15 OR #16 OR #17 OR #18 OR #19 OR #20 OR #21 OR #22 OR #23 OR #24 OR #25 OR #26

#28 #9 OR #27

#29 random*:ab,ti OR (clinical NEXT/1 trial*) OR 'health care quality'/exp

#30 #6 AND #28 AND #29

#31 random*:ab,ti OR placebo* OR (double NEXT/1 blind*):ab,ti

#32 #30 AND #31

**Cumulative Index to Nursing and Allied Health Literature (CINAHL) via EBSCO**

S1 (MH “Systemic Inflammatory Response Syndrome”) OR (AB “systemic inflammatory response syndrome”) OR (AB SIRS) OR (AB sepsis) OR (AB septic) OR (TI “systemic inflammatory response syndrome”) OR (TI SIRS) OR (TI sepsis) OR (TI septic)

S2 (MH Rehabilitation) OR (MH Exercise) OR (AB rehabilitation) OR (AB exercise) OR (AB training) OR (AB mobilization) OR (AB mobilisation) OR (AB “physical therapy”) (AB physiotherapy) OR (AB “occupational therapy”) OR (AB “electrical muscle stimulation”) OR (AB “cycle ergometer”) OR (AB bridging) OR (AB rolling) OR (AB “lying to sitting”) OR (AB marching) OR (AB ambulation) OR (AB “activities of daily living”) OR (AB ADL) OR (AB walking) OR (TI rehTIilitation) OR (TI exercise) OR (TI training) OR (TI mobilization) OR (TI mobilisation) OR (TI “physical therapy”) (TI physiotherapy) OR (TI “occupational therapy”) OR (TI “electrical muscle stimulation”) OR (TI “cycle ergometer”) OR (TI bridging) OR (TI rolling) OR (TI “lying to sitting”) OR (TI marching) OR (TI ambulation) OR (TI “activities of daily living”) OR (TI ADL) OR (TI walking)

S3 S1 AND S2

S4 ((MM“Randomized Controlled Trials”) OR (MM“Random Assignment”) OR (MH“Prospective Studies”) OR (MH“Multicenter Studies”) OR (MH “Double-Blind Studies”) OR (MH “Single-Blind Studies”) OR (MH “Triple-Blind Studies”) OR (MH “Placebos”)) OR ( random* OR (controlled AND (stud* or trial*)) OR ((blind* or mask*) AND (single or double or triple)))

S5 S3 AND S4

**PEDro**

Advance search

Abstract & Title: systemic inflammatory response syndrome OR SIRS OR sepsis OR septic

**The world health organization international clinical trials platform search portal (WHO ICTRP)**

Condition:  systemic inflammatory response syndrome OR SIRS OR sepsis OR septic
Intervention: Rehabilitation OR Exercise OR training OR mobilization OR mobilisation OR physical therapy OR physiotherapy OR occupational therapy OR electrical muscle stimulation OR cycle ergometer OR ambulation OR activities of daily living OR ADL OR walking
