# Supplementary material for: Rehabilitation for patients with sepsis: A systematic review and meta-analysis
Source: PLoS One. 2018 Jul 26;13(7):e0201292. doi: 10.1371/journal.pone.0201292 (PMC6062068; doi:10.1371/journal.pone.0201292)
Supplement: S1 Table — (DOCX) [file pone.0201292.s003.docx]

Characteristics of ongoing studies

| Trail name or title | Physical therapy during the early course of sepsis is safe and preserves skeletal muscle mass. |
| --- | --- |
| Methods | Study design: RCT  Country: Belgium |
| Participants | Inclusion criteria: Adult patients admitted with the diagnosis of severe sepsis |
| Interventions | Additional two times 30 min of passive/active cycling exercise |
| Outcomes | Skeletal muscle biopsy and electrophysiological testing, muscle histology, biochemical and molecular analyses of anabolic/catabolic and inflammatory signaling pathways, hemodynamic parameters and patients’ perception |
| Starting date | Unclear, but the trial was finished. |
| Contact information | Laterre, P. F.  email  [pierre-francois.laterre@uclouvain.be](mailto:pierre-francois.laterre@uclouvain.be) |
| Notes | Although some trial data are published in the Hichman (2017) abstract (Annals of Intensive Care, Volume 7, Issue 1, pp. 205-206 - published 2017-01-01), this trial is not published in full in any of the identified publications, and the author answered that l paper including length of stay, adverse events and mortality would be submitted soon. |

| Trail name or title | Early rehabilitation in critical care (eRiCC): functional electrical stimulation with cycling protocol for a randomised controlled trial. |
| --- | --- |
| Methods | Study design: RCT  Country: Australia |
| Participants | Inclusion criteria:  ≥18 years, expected MV>48 h with diagnosis of sepsis or severe sepsis as deﬁned by ACCP Consensus Conference Criteria, predicted ICU LOS ≥4 days.  Exclusion criteria:  known primary systemic neuromuscular disease or intracranial process at admission, lower limb amputation/s, unable to assess premorbid physical outcome measures due to condition impairing mobility, assessed by medical staff as approaching imminent death or withdrawal of medical treatment within 36 h, pregnancy, BMI>40, presence of external ﬁxator or superﬁcial metal in lower limb, open wounds or skin abrasions at electrode application points, presence of pacemaker or implanted deﬁbrillator, transferred from another ICU after >2 days of consecutive MV, platelets<40 000 and INR>1.6 (for biomarker subgroup). |
| Interventions | Functional electrical muscle stimulation-assisted supine cycling on one leg　while the other leg undergoes cycling alone |
| Outcomes | Muscle mass (quadriceps ultrasonography; bioelectrical impedance spectroscopy), muscle strength (Medical Research Council Scale, hand-held dynamometry) and physical function (Physical Function in Intensive Care Test, Functional Status Score in intensive care, 6 min walk test), biochemical/histological analyses of collected muscle, urine and blood samples |
| Starting date | May, 2012 |
| Contact information | Parry SM  email [selina.parry@austin.org.au](mailto:selina.parry@austin.org.au). |
| Notes | The Protocol is published (BMJ Open. 2012 Sep 13;2(5). pii: e001891. doi: 10.1136/bmjopen-2012-001891. Print 2012.) |

| Trail name or title | Sepsis Trial of Early Physical Therapy Outside the ICU: A Pilot Feasibility Study |
| --- | --- |
| Methods | Study design: RCT  Country: United States |
| Participants | Inclusion criteria:  Any English-speaking patient over the age of 18 who is currently admitted to a general care floor under the care of the Medicine Faculty Hospitalist Service at the University of Michigan Health System and has severe sepsis is eligible to take part in this study.  Exclusion criteria:  Subjects must be deemed by the study team to give informed consent by being able to state the basic purpose of the study. Consent by a legally authorized representative (LAR) will not be sought. |
| Interventions | Additional physical therapy |
| Outcomes | Change from baseline ambulation, activity of daily living, instrumental activities of daily living, discharge location, ICU transfer rate, length of stay, employment status, post-hospitalization physical function |
| Starting date | April 2014 |
| Contact information | Jeffrey Rohde, University of Michigan  https://clinicaltrials.gov/ct2/show/record/NCT02159222 |
| Notes | Study ID: NCT02159222 |

| Trail name or title | Early Mobilisation in Intensive Care Unit : Interest of Cyclo-ergometry in Patients With Septic Shock |
| --- | --- |
| Methods | Study design: RCT  Country: France |
| Participants | Inclusion criteria:  Patient hospitalized in ICU, septic shock diagnosed more than 24 hours before inclusion, patient hemodynamically stable, before the 72^th^ hour following the diagnosis of septic shock, mechanical ventilation by tracheal intubation, patient sedated with a RASS score inferior or equal to -2, age ≥ 18 years, BMI ≤ 40 kg / m², Informed patient having signed the consent, effective contraception in women of childbearing age (pregnancy test by B-HCG will be done; for menopausal women, a diagnosis of confirmation must be obtained).  Exclusion criteria:  BMI> 40 kg / m², patient reduced by one or two lower limbs, rheumatological pathology, trauma or surgery of the lower limbs, pelvis or spine, resulting of any limitations of the range of motions or strict immobilization, brain-injured patient and / or medulla injured, hemodialysis continues with a femoral catheter without possibility of changing the catheter location, moribund patient, stop or limitation of active therapeutics' decision, contraindications to standard physical therapy or the cyclo-ergometer, untreated orthopedics: deep vein thrombosis of the member concerned, dermatological: severe lesions or complex dressings in the sector concerned, patient with extracorporeal membrane oxygenation (ECMO), pregnant or breastfeeding women, patient participating in another trial with the same main objective. |
| Interventions | Early mobilization with cycloergometer |
| Outcomes | Number of days between hemodynamic stability and ICU discharge, number of days between hemodynamic stability and the removal of sedation, number of days under mechanical ventilation (invasive and noninvasive) during hospitalization in ICU |
| Starting date | December 2016 |
| Contact information | Gaétan BEDUNEAU, MD  https://www.smartpatients.com/trials/NCT02872792 |
| Notes | Study ID: NCT02872792 |
